# Water in Relation to Obesity

**Published:** 1889-05

**Authors:** 


### WATER IN RELATION TO OBESITY.

Dr. Lorenzen, of Erlangen, has been discussing the influence of liquids on obesity. The first experiment was made on himself. For a period of nine years he drank a large quantity of Erlanger beer daily. During four years of the period the daily quantity consumed amounted to 10 litres, or 2 gallons 1½ pints, or about 22 pounds weight; during the remainder of the period the quantity ranged from 5 to 7 litres in addition to 1 litre of wine. In this way he succeeded in increasing his bodily weight by 78 pounds, and the usual unpleasantness of obesity made its appearance. On shutting off the liquids his weight fell 14 pounds in seven days. If, however, more water was taken, but without alcohol, the weight increased again. Within five weeks he reduced him himself to the extent of 23 pounds, the chest measurement diminished by 7 ctm., and that of the abdomen by 13 ctm, and the difficulties attending respiration disappeared. Similar experiments carried out on colleagues, who were likewise heavy men, had similar results. The disappearance of fat on withholding fluids he endeavors to explain on the hypothesis that the cells whose province it is to decompose albumen, when a large quantity of fluid is taken, now expend part of their energy in the combustion of fat. The fat they consume is replaced by fat from the tissue.—*The London Medical Press.*

